# Commentary: Effects of Whole Body Electrostimulation Associated With Body Weight Training on Functional Capacity and Body Composition in Inactive Older People

**DOI:** 10.3389/fphys.2021.735818

**Published:** 2021-09-03

**Authors:** Alexandre Lopes Evangelista, Angelica Castilho Alonso, Raphael Ritti-Dias, Bruna Massaroto Barros, Cleison Rodrigues de Souza, Tiago Volpi Braz, Danilo Sales Bocalini, Julia Maria D'andréa Greve

**Affiliations:** ^1^Laboratório de Fisiologia e Bioquímica Experimental, Centro de Educação Física e Esporte, Universidade Federal do Espirito Santo, Vitoria, Brazil; ^2^Programa de Mestrado Ciências do Envelhecimento, Universidade São Judas Tadeu, São Paulo, Brazil; ^3^Programa de Pós-Graduação em Ciências da Reabilitação, Universidade Nove de Julho, São Paulo, Brazil; ^4^Laboratório de Avaliação do Movimento Humano, Universidade Metodista de Piracicaba, Piracicaba, Brazil; ^5^Departamento de Ortopedia e Traumatologia, Universidade de São Paulo Faculdade de Medicina, São Paulo, Brazil

**Keywords:** older adults, electrostimulation, physical function, body composition, functional fitness

First, we would like to thank the authors for their interest in this study, and in fact, the use of electrical stimulation has become a promising strategy, with interesting outcomes regarding sports approaches (Filipovic et al., 2016, 2019) and health considering different pathological conditions (Wittmann et al., 2016; André et al., 2021). Thus, in agreement with Pano-Rodriguez et al. (2019), we supported and suggested that more studies, such as randomized clinical trials and meta-analyses, should be carried out with older adults so that the method can be considered robust and its different forms of application can reach society coherently and positively.

Specifically considering the points indicated by Marocolo et al. (2021), before we received this comment, we had already requested the corrections to Table 2, according to the version of the Corrigendum published on May 20, 2021 (Evangelista et al., 2021). The authors mentioned that even in the version of the Corrigendum, there are still errors of 2.0 and 0.4 kg in the variable lean mass (according to their calculations) for the control and body weight associated with whole-body electrostimulation (BW+WB-EMS) groups, respectively. The calculations performed by the authors possibly used the average of our sample and not individual values, hence the divergence. In addition, the calculations made by the InBody230 equipment using bioimpedance have their predictive equations, which may explain small variations in the calculation of lean mass, including a standard formula probably used by Marocolo et al. (2021). It is also important to make clear that in this first part of the comment, the authors are reinforcing errors that had been corrected (Evangelista et al., 2021).

Marocolo et al. (2021) were correct in the notes regarding the correlations of the change in the vastus lateralis muscle with the 6-min walk distance (Figure 4C) and chair stand (Figure 4D). We made a mistake when writing the title of the axis of Figures 4C,D. Changes in the thickness (mm) of vastus lateralis should be on the *x*-axis. Thus, the range from −100 to 500 is relative to the distance in the 6-min walk test. As you can see, the individual range of changes in the thickness of vastus lateralis was 1.0–9.1 mm. As the comment by Marocolo et al. (2021) gave a lot of attention to this variable, we decided to describe the individual data of vastus lateralis muscle change in 10 subjects in the BW+WB-EMS experimental group in the response. The ascending order of the change was 1.0, 1.6, 2.7, 3.8, 4.0, 4.2, 5.4, 6.3, 6.5, and 9.1 mm (mean = 4.5 mm). Furthermore, in this study, the typical error measurement (TEM) of the thickness of vastus lateralis measured by B-mode ultrasound was 0.41 mm. These values of change in 6–8 weeks and the TEM of vastus lateralis corroborate the literature (Oranchuk et al., 2019, Katsura et al., 2019). We would like to point out that despite the error in the title of the axis in Figures 4C,D, the mean difference (MD) and 95% CI of the changes in the thickness of vastus lateralis had already been reported in the manuscript, specifically in the 6th line of the 2nd paragraph of the results “MD (95% CI) = 4.5 (1.7–4.3 mm) in the BW+WB-EMS group.” Thus, we agreed with Marocolo et al. (2021) that a change of 400 mm in a subject in 6 weeks is unrealistic for the thickness of vastus lateralis; however, this was not the case for the research results. We had already noticed the error and sent a new Corrigendum to the journal on May 25, 2021, approved on May 31, 2021. In addition, we would like to emphasize that the presentation of figures in univariate scatter plots as performed in this study makes it easier to detect gross violations of statistics, which reinforces the impartiality in the treatment of data. This is especially important for investigators who use parametric analysis to compare groups in small studies (Weissgerber et al., 2015).

We also recognized the discussion proposed in the study by Feng et al. (2014) on data transformation into logarithms. However, it is worth noting that the authors themselves recognize the importance of log transformation in the study by Feng et al. (2013): “We agree with Drs. Bland, Altman, and Rohlf that ‘Log-transformation is a valuable tool in the analysis of biological and clinical data' (Bland et al., 2013). We are by no means against the use of log transformation.” Notably, log transformations are typically applied to data that are expected to be log-normally distributed or at least to follow positively skewed distributions, yet their simulations are applied to data from a symmetrical, uniform distribution (Bland et al., 2013). This characteristic applies to the study data for all variables. On the presentation of raw or log values, we considered that the transformation only serves to make inferences, that is, to test the study hypotheses (Hopkins et al., 2009). Therefore, the final results were presented using the original scale, considering the difference between the geometric and arithmetic mean for all variables. This is a procedure that has already been mentioned in other studies (Aubry et al., 2014; Plews et al., 2014; McNamara et al., 2015; Jackman et al., 2019).

The age of the participants (mean age 75.1 ± 6.58 years) was included in the Corrigendum approved on May 31, 2021. For both groups, the training sessions included a 5-min warm-up (2 min of mobility exercises and 3 min of low-intensity calisthenics based on a rating of the perceived exertion scale, with the intensity maintained between 4 and 5).

After the warm-up, as already described in the original article, the exercise sessions were performed, including eight exercises using body weight, with two sets each and eight repetitions per set. An interval of 30 s was given between sets and 48–72 h between training sessions. The following exercises were applied: standing fly, squat, neutral pull, standing unilateral knee flexion, lateral raise, simultaneous curl, simultaneous elbow extension, and standing abdominal. The cadence of repetitions was conducted in a controlled fashion, with concentric and eccentric actions of ~2 and 4 s, respectively, with a total repetition duration of ~6 s.

Subjects in the control group were asked to contract the muscle at maximum intensity through the full range of motion, without the use of external load. The subjects in the BW+WB-EMS group were asked to rate the average intensity of the exercise session and the regional intensity of the EMS on a rating of the perceived exertion scale, and the intensity was maintained between 7 and 8 throughout the experimental protocol. Research staff, certified in the use of the technology, supervised all training sessions, provided verbal encouragement, and ensured that the subjects performed the correct number of sets and repetitions with the correct exercise technique.

It is worth emphasizing that the focus of this study was to verify the effectiveness of using the WB-EMS on functional and anthropometric parameters (body composition) in older adults and not to praise training methods and strategies. However, considering the position of the authors of the commentary the comparison between traditional strength training methodologies and the use of WB-EMS is still not clear in the literature. Thus, to our knowledge, few studies have been dedicated to investigating this purpose. Kemmler et al. (2016) observed comparable or at least similar increases in muscle parameters after 16 weeks of WB-EMS compared with high-intensity resistance training (RT). Šarabon et al. (2020), through a meta-analysis, indicated that both traditional strength training and WB-EMS are effective for increasing muscle strength; however, at present, due to limitations in studies with WB-EMS, RT, or combinations of RT and WB-EMS are probably the most reliable ways to improve the muscle strength and functional performance, while the best approach for increasing the muscle mass in older adults remains open to further studies.

In this context, some research proposals are similar to this study. Oliveira et al. (2021) pointed out that electrostimulation (EMS) is an innovative training technology, efficient in terms of time and physical function. In addition, Frontiers in Physiology published a Research Topic on the use of WB-EMS (https://www.frontiersin.org/research-topics/7876/whole-body-electromyostimulation-a-training-technology-to-improve-health-and-performance-in-humans#articles). Furthermore, some studies consider that WB-EMS could be an option for training, mainly due to the positive outcomes in body composition and muscle strength (Kemmler et al., 2010a,b; Kemmler et al., 2012; Pano-Rodriguez et al., 2020) and for presenting interesting results with regard to adherence and low dropout rates in older adults (Pano-Rodriguez et al., 2020).

We agreed that the modality is onerous nowadays; however, in agreement with Kemmler et al. (2016), we believed that over time WB-EMS will become more viable and cost-effective. We believed in this mainly because some companies working with electrostimulation already use wireless technology and clothing made from more affordable materials. In addition, there has been a clear increase in gyms using electrostimulation in Brazil in recent years. As an example, currently, ~44 units offer the modality only in the city of São Paulo (https://www.gympass.com/academias/em/sp/sao-paulo/com-aula-de/eletroestimulacao). Therefore, this is not a sufficient reason to include it as a limitation of this study, especially because we did not aim to perform any cost-effectiveness analysis.

Finally, we would like to thank the authors for the discussion and attention addressed to this study.

## Author Contributions

All authors contributed to the preparation of the entire research project, writing, selection of participants and collection of data, data review and analysis, editing, statistical analysis, discussion of results, and execution of the revision.

## Conflict of Interest

AE declares a conflict of interest for working as a scientific adviser for XbodyBrazil. The remaining authors declare that the research was conducted in the absence of any commercial or financial relationships that could be construed as a potential conflict of interest.

## Publisher's Note

All claims expressed in this article are solely those of the authors and do not necessarily represent those of their affiliated organizations, or those of the publisher, the editors and the reviewers. Any product that may be evaluated in this article, or claim that may be made by its manufacturer, is not guaranteed or endorsed by the publisher.
